# FACTORS CONTRIBUTING TO GROWING DEPRESSION AMONG ELDERS IN SUB-SAHARAN AFRICA

**DOI:** 10.1093/geroni/igad104.0409

**Published:** 2023-12-21

**Authors:** Margaret Adamek, Messay Kotecho

**Affiliations:** Indiana University, Indianapolis, Indiana, United States; Addis Ababa University, Sadist kilo, Adis Abeba, Ethiopia

## Abstract

While growing research has addressed physical ailments and communicable diseases in the older adult population in Sub-Saharan Africa, little research has investigated mental health conditions such as depression and anxiety among older Africans. In many African nations, mental health diagnoses are not acknowledged let alone well understood by the general public. Little has been documented about the extent of depression among older adults in Sub-Saharan Africa and the factors contributing to depression among older African adults. This study used secondary sources and a synthesis of recent empirical literature to examine and document the extent of depression among older adults in Sub-Saharan Africa and the contributing factors to geriatric depression. Sources indicate that mental health symptoms of Africans are often considered the work of evil spirits or may be overlooked by families. Available studies report rates of geriatric depression ranging from 37.8% in Ghana, 40% in South Africa, 41.2% in Ethiopia, and up to 44% in Tanzania. Older women in Sub-Saharan Africa have higher rates of depression than older men. Contributing factors to depression include lack of formal education, chronic illness, unstable income, and waning social supports. With an estimated 2 out of 5 older adults in Sub-Saharan Africa experiencing depression, interventions are needed to address the factors contributing to depression among older adults as well as culturally relevant interventions to ameliorate current symptoms. Interventions common in Global North nations such as anti-depressant medication or private therapy may not be appropriate for older African adults struggling with depression.

